# Beyond Immunity: Macrophages as Regulators of Vertebrate Morphogenesis

**DOI:** 10.3390/cells15151330

**Published:** 2026-07-24

**Authors:** Goretti Moran, Cristina Duarte-Olivenza, Juan M. Hurle, Juan A. Montero, Carlos I. Lorda-Diez

**Affiliations:** Department of Anatomy and Cell Biology and IDIVAL, University of Cantabria, 39011 Santander, Spain; goretti.moran@unican.es (G.M.); cristina.duarte@unican.es (C.D.-O.); juan.hurle@unican.es (J.M.H.); monteroja@unican.es (J.A.M.)

**Keywords:** apoptosis, phagocytosis, embryonic macrophages, macrophage plasticity, morphogenesis

## Abstract

Research over the last decades have demonstrated that macrophages, considered canonical players of the immune system, also perform many other important biological functions in adult organisms. These properties have stimulated extensive research, particularly given their importance in human pathologies, including cancer. However, despite significant advances in our understanding of macrophage functions in adult organisms and diseases, their roles in embryonic systems remain comparatively underexplored. Owing to their complex developmental origin and the lack of pronounced phenotypes in embryos subjected to either spontaneous or experimentally induced macrophage ablation, macrophages have often been considered as a largely passive scavenger population associated with programmed cell death during organ and tissue remodeling. Here, we highlight key findings regarding macrophage functions during vertebrate morphogenesis. We emphasize the differences in phenotypic outcomes following macrophage ablation in adult organisms, embryos, and models of organ regeneration. Currently, the remarkable plasticity of the macrophage lineage complicates the identification of specific trophic functions involved in organ morphogenesis. Developing new approaches that improve the efficiency of macrophage ablation models, along with implementing complementary gain-of-function strategies, will contribute to a deeper understanding of the role of macrophages during embryonic development.

## 1. Introduction

In this review, we critically examine the current knowledge of the role of macrophages in vertebrate embryonic development. We highlight that the morphological abnormalities associated with macrophage depletion or dysfunction are generally more pronounced during postnatal development and in models of adult epimorphic regeneration than during embryogenesis, despite the central role of macrophages in programmed cell death underlying the morphogenesis of most organs. We will address aspects such as the persistence of residual macrophage populations in loss-of-function models, the relevance of macrophage functional plasticity, and the ability of non-professional phagocytes to functionally compensate for macrophage deficiency.

## 2. Conceptual Framework and Development

Macrophages are effector cells of innate immunity that play a central role in host defense through the recognition, engulfment, and degradation of dead cells as well as harmful endogenous or exogenous agents. Cells exhibiting macrophage-like phagocytic properties are evolutionarily conserved and are found across all metazoan species [1,2,3]. Beyond their canonical role in debris removal, macrophages in vertebrates perform a broad spectrum of physiological functions [4,5,6,7,8,9]. They contribute to tissue growth and organ maturation during the postnatal period [10], are essential for maintaining the structural organization and functional integrity of specialized systems such as the cochlea in the auditory apparatus [11], and regulate the sensorimotor function of muscle spindles of skeletal muscle [12,13], as well as bioelectrical conduction in the heart [14]. Furthermore, macrophages have significant clinical relevance in the context of solid tumors, where they influence therapeutic responses and modulate the metastatic potential of cancer cells [15,16,17,18]. It is important to emphasize that in embryonic vertebrates, the phagocytic activity of macrophages is shared with other cell types that have been termed “non-professional” phagocytes [19,20], in contrast to “professional” macrophages (see below for details).

**Figure 1:**
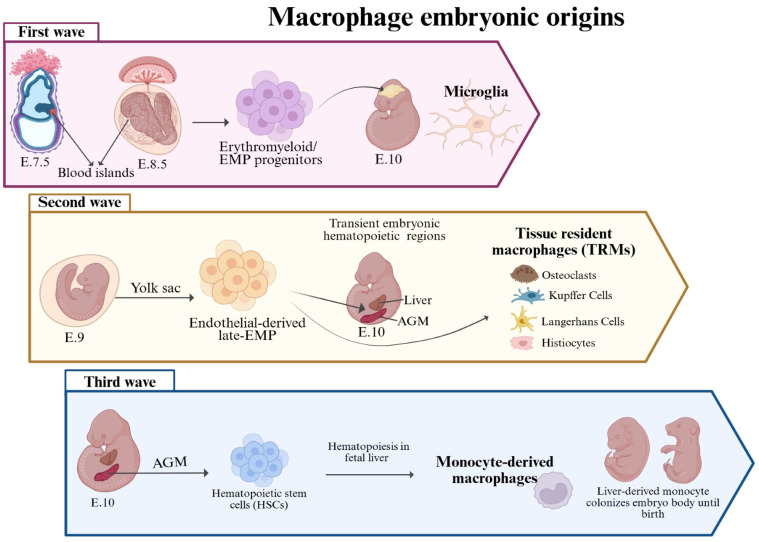
Sequential formation of macrophages in the developing mouse. Created in BioRender. https://BioRender.com/aety9js (accessed in 15 July 2026).

Macrophage ontogeny occurs during vertebrate development following a complex spatial–temporal sequence (see Figure 1) that has been most extensively characterized in mammals through lineage tracing experiments (reviews in [8,9,21,22]). The earliest macrophages arise at very early developmental stages within the blood islands of the yolk sac, through the generation of erythromyeloid progenitors [23,24,25]. These progenitors constitute the first wave of macrophages that colonize the embryonic central nervous system and give rise to microglia [26]. Later in embryonic development, a second wave of progenitors arises in the yolk sac, referred to as endothelial-derived late-EMPs, which disseminate throughout embryonic tissues to give rise to the so-called “tissue-resident macrophages”. This population includes, among others, Kupffer cells in the liver, lung alveolar macrophages, histiocytes in the connective tissues, osteoclasts in bone primordia, etc. These tissue-resident macrophages exhibit trophic functions and persist, being active and self-renewing during fetal and postnatal periods.

At subsequent stages of embryonic development, as well as during the fetal period, transient hematopoietic sites emerge within the embryo, giving rise to macrophage populations with distinct functional and molecular profiles [22,27,28]. These regions include the fetal liver [29] and the confluence between the ventral aortic wall, the gonadal primordium, and the mesonephros (AGM; [30]). The third wave of macrophage production arises from hematopoietic stem cells (HSCs) located within these hematopoietic regions. The final phase of macrophage development depends on the bone marrow, which, following colonization by fetal liver-derived progenitors, becomes responsible for macrophage production during the perinatal period and throughout postnatal life via monocyte differentiation [29].

In addition to the canonical sources of macrophages, an alternative origin for a subset of macrophages in the embryonic heart has been suggested [31,32,33]. It has been proposed that these macrophages differ from those generated in the yolk sac or at major hematopoietic sites (AGM and liver) and could represent an evolutionarily conserved hematopoietic pathway also present in Drosophila and zebrafish [34]. According to this proposal, cardiac macrophages in murine embryos would originate from the endothelial layer of the heart (endocardium), persisting into adulthood, and are capable of giving rise to not only macrophages but also cardiomyocytes and endothelial cells [33]. To our knowledge, with the exception of the placental vasculature [35], the existence of alternative origins of macrophages in other embryonic organs has not been reported.

## 3. Genetic Mechanisms Governing Macrophage Differentiation

CSF1R (*Colony-Stimulating Factor-1 Receptor*; *c-fms* gene) is the main signaling receptor controlling activation, differentiation, and maintenance of macrophages. It reflects the developmental dependence of macrophage progenitors for two signaling ligands: Colony-Stimulating Factor 1 (CSF1) [36,37,38] and Interleukin-34 (IL-34) [39,40]. High expression levels of *CSF1R* are widely considered a specific marker of the macrophage lineage in vertebrates, although expression is also observed in placental trophoblast [41,42,43] and in a limited number of other normal or pathologic tissues [44]. However, although genetic silencing of *CSF1R* gene results in a massive decrease in the macrophage population in rodents, some residual macrophages persist in tissues such as the splenic red pulp, the liver, the lungs, and the intestine [10,45].

Besides CSF1R, PU.1 (Sfpi-1 proto-oncogene) is a pivotal transcription factor in the development of macrophages in embryonic and adult hematopoietic regions. In these hematopoietic sites, the differentiation of the different blood cell lineages is regulated by the balanced activities of lineage-determining transcription factors [46,47], and silencing of the PU.1 gene in mice results in the absence of macrophages and B cells [48,49].

## 4. Macrophage Phenotypic Diversity

It is important to note that although macrophages are often described as a discrete and well-defined cell lineage, their distribution across diverse tissues and their exposure to distinct microenvironmental conditions give rise to pronounced phenotypic and transcriptional heterogeneity [28]. This heterogeneity underlies the acquisition of functional properties, commonly referred to as “polarization states.” Accordingly, macrophage secretory profiles differ according to their polarization state, which is shaped by environmental cues that activate specific transcriptional programs [50,51] (see reviews in [6,36,52]).

**Figure 2:**
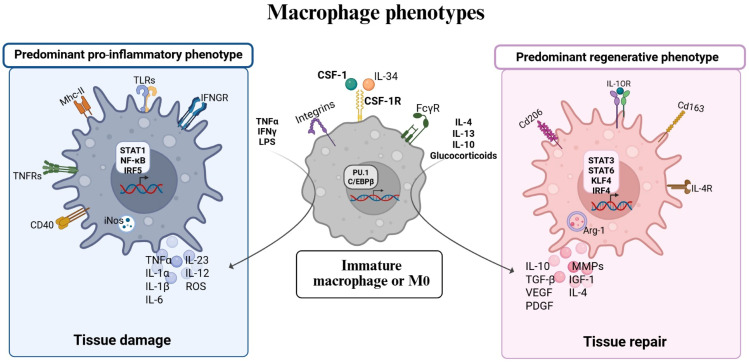
Schematic representation of molecular and transcriptional regulation of extreme functional subtypes of the macrophage’s spectrum. Created in BioRender. https://BioRender.com/mvb3tzh (accessed in 15 July 2026).

Two extreme functional/polarization states of macrophages are commonly recognized (see Figure 2): the “pro-inflammatory” state (also referred to as “classically activated” or “M1” macrophages) and the “anti-inflammatory/pro-regenerative” state (also referred to as “alternatively activated” or “M2” macrophages). Canonically, M1 macrophages secrete pro-inflammatory cytokines, including interleukin-1α (IL-1α), IL-1β, IL-6, IL-12, IL-23, and TNF-α, thereby promoting ROS-mediated tissue damage. In contrast, the prototypical M2 macrophages are characterized by high phagocytic capacity and the secretion of anti-inflammatory cytokines and growth factors including IL-4, IL-10, and TGF-β, which promote tissue repair, angiogenesis, and fibrosis (reviewed by [53]). Nevertheless, accumulating evidence indicates that macrophages comprise a highly heterogeneous population with diverse transcriptional profiles and functions in both developing and adult organisms. Consequently, the M1/M2 paradigm should be regarded as a simplified conceptual framework rather than a strict biological classification, serving only as a tentative indication of the predominant functional state of macrophages.

As mentioned above, macrophages play important trophic and pro-regenerative functions via the production of a secretome that contains components also present in senescent cells [54,55,56]. The delivery of these factors may be mediated by exosomes and operate through paracrine cell communication (see reviews in: [57,58]).

The macrophage secretome includes non-coding RNAs [44]; a variety of growth factors, including Transforming Growth Factors-β (TGF-βs) [59], WNTs [60], IGFs [61,62], EGF [63], FGFs [64], HGF [65], VEGF [61,66,67], PDGF [68] as well as extracellular matrix molecules (elastases, as well as metalloproteinases such as ADAMTS MMP2, MMP7, MMP9, and Netrin-1, among others [26,69,70]); and, small immunomodulatory molecules (cytokines and chemokines; see above).

However, a spectrum of macrophage functional states is associated with distinct transcriptional profiles that may include combinations of M1 and M2 markers [71]. In addition, inter-species transcriptional differences between the same macrophage populations are also observed [72]. This may explain why the precise correlation between macrophage transcriptomal profiles and functional states has not been fully established [4,50,52].

## 5. Structural and Functional Plasticity of Macrophages

Macrophages are characterized by their functional and developmental plasticity, which enables them to adapt to the surrounding environment by recognizing different signals. At the morphological level [73], immature macrophages are characterized as rounded cells with weak immunoreactivity for the macrophage marker F4/80 (an orphan receptor involved in cell adhesion; *Emr1* gene) along with a limited number of lysosomes [73,74]. Upon initiation of phagocytic activity, these “activated” macrophages [52] undergo marked changes, including an increase in cell size, increased F4/80 positive immunolabeling, and the presence of prominent heterophagic vacuoles containing lysosomal enzymes.

Following their incorporation into embryonic tissues, macrophages engage in dynamic interactions with neighboring cells, thereby establishing functional niches that determine both their number and phenotypic profile [51,75,76,77]. The niches are shaped by tissue-derived signals including metabolites, growth factors, cytokines, and cell–cell interactions (see Table 1). This process appears to proceed through a multistep sequence, initially driven by metabolic cues via the polyamine–hypusine pathway [78] and the subsequent activation of specific transcription factors [36], which ultimately determine macrophage identity [75]. These regulatory inputs modify the epigenetic and transcriptional profiles and control the population size by influencing self-renewal and survival [76,79,80,81].

Functional plasticity is also observed following the administration of exogenous macrophage progenitors or bone marrow cells into macrophage-deficient hosts. Under these conditions, the donor progenitors are capable of reconstituting endogenous macrophage populations [10,82,83,84,85] while reassuming the specialized functional characteristics of the resident populations they replace [48,86].

**Table 1:** Signaling mechanisms responsible for maintenance of macrophage niches in vertebrates.

Macrophage Niche Signaling		
Molecule	Function	Reference
CSF1 (M-CSF)	Main CSF1R ligand that regulates differentiation, survival, proliferation, and maintenance of macrophages. Promotes M2-like regenerative phenotype.	[36]
IL-34	CSF1R alternative ligand, important for the maintenance of homeostasis in microglia and Langerhans cells.	[87]
CSF2 (GM-CSF)	Promotes M1-like inflammatory phenotype.	[76]
TGFβ	Acts as an “architect” of the niche, maintains cell identity, promotes “quiescence,” and coordinates tissue repair after injury.	[88]
Growth factors	Growth factors released by macrophages (FGF, PDGF, VEGF, EGF, IGF-1, or HGF), promote growth and regeneration of adjacent tissues.	[67]
Integrins	CD11b and VLA-4 allows the adhesion of macrophages to the extracellular matrix and fibroblast.	[76]
Matrix remodeling molecules	Elastases, ADAMTs, and MMPs that modulate the extracellular matrix.	[26]
Transcription factors	Such as PU.1; GATA6; RUNX3; MAFB; or PPARγ. Promote specific tissue resident macrophage identity. Activated by retinoic acid, Notch ligands, TGF-β, CSF1, CSF2, or IL-34.	[75]
Extracellular vesicles (EVs)	EVs contain cytokines, growth factors, proteins, and miRNAs that allow for the reprogramming and polarization of macrophages.	[89]

## 6. Macrophages Phagocyte Dead Cells and Their Remnants in Vertebrate Embryos

During vertebrate development, the best recognized function of macrophages is the removal of dead cells by phagocytosis, a process that has been termed “efferocytosis” since it results in the definitive elimination of dead cells [90]. This mechanism requires macrophages to identify “eat-me signals” on the surface of dying cells, while living cells may produce “don’t eat-me signals” that protect themselves from being phagocytosed [91] (see review in [92]). As previously mentioned, phagocytosis is not an exclusive function of macrophages (see review in [93]). Within the embryo, cells including immature myocardial cells [19,94] or neuroblasts [20,95] have been shown to phagocytose dead cells. The term of “non-professional phagocytes” has been proposed to designate these non-specialized phagocytes. Whether non-professional phagocytes are activated by the same or by different signals employed by macrophages is not known. In the developing embryonic limb, it has been proposed that reduced expression and regulation of macrophage phagocytosis genes, such as the *Abca1 transporter* gene (homologue of the ced-7 phagocytosis gene of the worm *Caenorhabditis elegans*; [96]), may explain the lower efficiency of non-professional phagocytes in the removal of dead cells compared to professional macrophages [49].

In non-apoptotic death processes involving plasma membrane rupture, the release of intracellular components into the extracellular space, including nucleic acids [97], facilitates their recognition by macrophages, which, in turn, induces a pro-inflammatory response. Conversely, apoptosis, which is the characteristic mode of cell death in the embryo, preserves considerable membrane integrity until advanced stages of degeneration. Distinct signaling mechanisms for the recognition of apoptotic cells by macrophages have been identified [98]. Among these signals [91], the redistribution and surface exposure of phosphatidylserine from the inner side of the cell membrane [59] is the best characterized. Notably, the apoptotic signal results in a preferential anti-inflammatory response by macrophages [99,100]. However, in developing systems, apoptotic cells undergo secondary necrosis if they are not promptly removed by macrophages [101], suggesting that the balanced activation of functionally distinct macrophages in embryonic areas of apoptosis may depend on the efficiency of the phagocytic process.

Macrophages must be recruited from regions of the embryo other than those where cells die (“come-get-me” or “find-me” signals; reviewed in [92,98]). Distinct macrophage chemoattractant signals delivered by apoptotic cells or their neighbors have been identified in vitro, including extracellular nucleotides [102], phospholipids released by the action of Caspase3 [103], and chemokines such as CX3CL1 (*Fractalkine*) [104] or CCL2 (MCP-1) [105,106,107]. In embryos, chimeric grafting experiments confirm the rapid and potent attraction of host macrophages to areas of cell death within grafted tissues [108]. The nature of the chemoattractant signals directing macrophages to embryonic areas of cell death remains uncharacterized (reviewed by [98]). However, although still lacking full experimental support, it is tempting to speculate that exosomes contribute as carriers of chemoattractant signals [57].

## 7. Macrophages Influence Cell and Tissue Dynamics Underlying Morphogenetic Events

Macrophages modulate fundamental cellular behaviors and extracellular matrix differentiation, thereby influencing the outcome of developing organisms, as well as tumor progression. These functions depend on the local release of components of the macrophage secretome, detailed above. Moreover, the nature of these secreted products varies according to macrophage phenotype, which is itself strongly influenced by microenvironmental signaling, thereby establishing complex feedback regulatory mechanisms.

Cell and molecular mechanisms employed by macrophages to regulate the activities of neighboring cells generally act in a coordinated manner and are highly context-dependent. The principal mechanisms are summarized below.

(1) Induction of cell death: Apoptosis induction is a well-established physiological mechanism through which macrophages directly regulate the survival of healthy cells. In developing systems, this function has been demonstrated in the regression of hyaloid vasculature and pupillary membrane of the developing eye, where macrophage-derived Wnt7b promotes programmed cell death [60,109]. In adult inflammatory conditions and cancer, macrophage-mediated pro-apoptotic activity largely depends on the production of reactive oxygen species (ROS) via cytotoxic cytokines, particularly TNF-α [110].
(2) Inhibition of cell proliferation: Macrophages suppress cell proliferation through the release of anti-proliferative mediators, including p21, TNF-α, interferon gamma, members of the TGF-β family, or nitric oxide (NO). The latter has been shown to inhibit cell proliferation in several cell types including T cells [111,112], vascular endothelial cells, epithelial tissues, and tumor cells [113,114].
(3) Promotion of cell survival and proliferation: The ability of macrophages to promote cell survival and proliferation is an evolutionarily conserved function that contributes to tissue homeostasis, regeneration, and repair. Macrophage-derived growth factors, including FGF2, TGF-α, IGF-1, VEGFA, and, in specific contexts, TGF-β isoforms, stimulate the proliferation of diverse cell populations. Representative examples include angiogenesis [115], regeneration of insulin-depleted pancreatic beta-cells [116,117], and neonatal cardiomyocyte regeneration [118].
(4) Regulation of cell migration: Macrophages regulate both the activation and inhibition of cell migration through the secretion of a complex repertoire of mediators and by modifying extracellular matrix (discussed below). This regulatory activity affects numerous target cell populations, including lymphocytes, epithelial and endothelial cells, fibroblasts, stem cells, and macrophages themselves. Examples include the regulation of stem cells behavior within the corneal limbal niche [119] and cardiac scar formation in adult ischemic hearts [120].
(5) Regulation of cell metabolism: Macrophages actively regulate cellular metabolism, particularly lipid metabolism, thereby modifying the local microenvironment and influencing the growth, differentiation, and functional state of neighboring cells [121]. These metabolic functions have important pathological implications in atherosclerosis [122] and in various tumor types [123].
(6) Extracellular matrix remodeling. Many products of the macrophage secretome are direct regulators of the extracellular matrix that function in cooperation with the above-described regulators of cell behavior. For example, remodeling of the extracellular matrix by metalloproteinases may subsequently promote the delivery of growth factors to regulate angiogenesis [124]. One of the most representative consequences of this effect occurs in developing systems involving epithelial/mesenchymal interactions that drive branching and growth of the epithelial component (i.e., mammary gland and bronchioalveolar system) [125]. Furthermore, in cancer, macrophage-mediated matrix remodeling has a profound influence on tumor progression [126].

### 7.1. Macrophages and Organ Morphogenesis

The morphogenetic role of cellular and molecular signals in embryonic models has traditionally been based on loss- and gain-of-function methodologies. As summarized below, the knowledge of the role of macrophages in developing systems is derived from genetic or chemical macrophage ablation approaches. In contrast, gain-of-function experiments remain scarce, with the exception of studies involving CSF1 treatment or the reintroduction of macrophage progenitors into organisms lacking them.

The genetic induction of macrophage-deficient individuals relies on the (spontaneous or experimentally induced) silencing of master macrophage genes (i.e., *CSF1/Csf1r* or *PU.1* genes). Under these conditions, the number of macrophages is drastically reduced, although in the case of CSF1/CSF1R axis disruption, it has been reported that a small macrophage population persists [41]. Given the enormous plasticity and regenerative potential of macrophages (reviewed above), this could be a limitation in the interpretation of findings derived from loss-of-function approaches. Therefore, the major question concerning the interpretation of phenotypes resulting from macrophage ablation approaches is as follows: Why are macrophages abundant in embryonic regions undergoing programmed cell death and tissue remodeling, yet macrophage-deficient embryos often show relatively mild gross morphological phenotypes compared with postnatal or regenerative models?

The phenotypic differences observed during postnatal and embryonic periods following macrophage ablation could be explained by different processes. It has been proposed a potential role for the activation of non-professional phagocytes [49]. However, they may also reflect the persistence of a residual macrophage population in combination with a greater macrophage plasticity in embryos than in adult organisms. This interpretation is further supported by the more pronounced morphogenetic defects observed during adult epimorphic regeneration under macrophage-depleted conditions.

### 7.2. Postnatal Period

The postnatal phenotype of mammals subjected to targeted gene disruption or spontaneous mutations affecting macrophage-related genes illustrates relevant functions of macrophages in organs that complete morphogenesis in the postnatal period (Supplementary Table S1). Although minor species-specific differences are observed among mice, rats, and humans, all share common features associated with macrophage dysfunction [10,127,128] and can be ameliorated by exogenous CSF1 administration that promotes growth and differentiation of macrophages [129].

Major postnatal morphological alterations reflect (i) the depletion of specific macrophage populations (microglia and osteoclasts); (ii) persistence of hyaloid vessels and pupillary membrane in the eye; (iii) impaired organ growth and differentiation resulting from alterations in extracellular matrix remodeling; and (iv) deficient development of pancreatic β-cells [130]. Additional alterations in various organs (lung, spleen, liver, skin, gut, cerebral cortex, etc.) include reduction of white adipose tissue, neuronal loss in the cerebral cortex, defective hematopoiesis, endocrine dysregulation, and impaired vascular growth, all of which accompany the anomalies observed in postnatal macrophage-depleted individuals [7,10,62,127,128,131,132,133,134].

Genetic disruption of the CSF1R/CSF1 axis is characterized by the absence of microglia, accompanied by deficits in neuronal connectivity that affect neural circuits such as the hypothalamic–pituitary–gonadal axis, which, in turn, leads to reproductive alterations [135,136]. However, the phenotype is less severe in individuals deficient in CSF1 than in those lacking CSF1R, due to the alternative signaling mediated by IL-34 [39]. Notably, central nervous system gross morphology remains largely preserved, with the exception of ventricular dilatation [5,137]. Heterozygous inactivation of the *Csf1r* gene in humans underlies “hereditary diffuse leukoencephalopathy with spheroids”, a condition attributed to microglia deficiency [138,139,140].

Another notable phenotype caused by alterations in the CSF1R/CSF1 axis is characterized by skeletal alterations and smaller body size. These defects arise from osteoclast dysfunction and affect bone growth and differentiation, including increased bone density, abnormalities in growth plates, and reduced bone marrow cavities in long bones collectively referred to as “osteopetrosis” [127,141]. Unlike the microglia defects, skeletal phenotypes are more severe after Csf1r gene inactivation than CSF1 deficiency [129]. Indeed, IL-34 deficiency results primarily in microglial and skin macrophage-associated phenotypes indicative of a primordial function of CSF1 in osteoclasts [142]. A species-specific feature observed in rats is a toothless phenotype, resulting from the inability of teeth to erupt through abnormally densified maxillary bones [143].

Disruption of the CSF1R/CSF1 axis also leads to alterations associated with extracellular matrix remodeling. These arise from impaired local production of matrix remodeling enzymes, growth factors, and/or cytokines due to the absence of macrophages. As a result, macrophage-dependent regulation of cell migration, growth, and differentiation is compromised in organs undergoing vascular remodeling, bud branching morphogenesis, or that complete morphogenesis in the postnatal period. In mammals, such defects are evident in multiple organs, including impaired growth and branching of the mammary gland during puberty and pregnancy [125,144,145,146], structural abnormalities in the pancreas with loss of insulin-producing β-cells [130,147], kidney weight reduction [148], and defective alveolar formation during postnatal lung development [149].

Alterations are also observed in other vertebrates such as the metamorphosing zebrafish, which offers a very illustrative example of how macrophages direct morphogenesis by regulating the behavior of neighboring cells. In this species, the ablation of macrophages during larval metamorphosis prevents the proper migration of melanophores required for the formation of the fish’s characteristic pigmented stripes [150].

The transcription factor PU.1 is essential for the differentiation of bone marrow-derived macrophages and underscores their hematopoietic origin. In contrast to the alterations caused by the disruption of the CSF1R/CSF1 axis, phenotypes resulting from PU.1 gene disruption are less severe. Among the phenotypes resulting from PU.1 gene silencing, the persistence of hyaloid vessels is particularly noteworthy. In mammals, final maturation of the visual organs takes place in the perinatal or early postnatal period and requires the remodeling of the pupillary membrane and hyaloid vessels to permit light transmission to the retina. These processes are impaired in PU.1-deficient mice supporting the involvement of macrophages in this important morphogenetic process [60]. Furthermore, the malformation is replicated in mice transfected with the diphtheria toxin gene targeted to macrophages [151] and by transcorneal injection of clodronate encapsulated in liposomes, which kills macrophages upon ingestion [109]. These findings support a role for macrophages in promoting cell death. Mechanistically, induction of cell death by macrophages in the eye was shown to be mediated by the delivery of WNT7b [60]. However, although visual deficiencies due to compression of the optic nerve are observed in osteopetrotic mice [152], this phenotype was not reported in murine models [41,128] or humans deficient in *Csf1r* gene [45].

### 7.3. Embryonic Period

In vertebrate embryos, macrophages are widely distributed throughout the embryonic body [5,108], showing massive accumulations in the areas of programmed cell death, where they remove apoptotic cells and cell remnants [55,90,153]. Considering that programmed cell death is a pivotal mechanism of morphogenesis [154], accounting for removal of transient evolutionary structures (“phylogenetic cell death”, i.e., mesonephros disappearance in mammals), the sculpting of the shape of embryonic organs (“morphogenetic cell death”, i.e., digits in species with separated digits), or the removal of excess progenitor cells during tissue structural differentiation (“histogenetic cell death” reviewed in [154,155,156]), it might be expected that macrophage ablation would result in severe gross anatomical malformations in the embryo.

However, in contrast to the pronounced alterations observed during the postnatal period, embryonic development (excluding the ablation of microglia described above during the postnatal period) is comparatively much less affected after genetic disruption of the CSF1R/CSF1 axis or the transcription factor PU.1 [48,49]. Under these gene-deficient conditions, gross morphological abnormalities in organs sculpted by programmed cell death [41] are not observed. The absence of gross embryonic phenotypes in macrophage-deficient embryos may reflect compensatory mechanisms. Among these, this may include the reprogramming of undifferentiated nonspecific progenitors in the embryonic period to substitute the absent macrophages or the capacity of other embryonic cell populations to assume macrophage functions (“non-professional phagocytes”) in the absence of local CSF1R-positive or PU.1-positive tissue-resident macrophages [49].

The involvement of macrophages in the development of other embryonic organs, including the kidney, has been inferred from their distribution within its embryonic primordia and from experimental manipulations using CSF1 treatments or neutralizing antibodies against CSF1 administered to cultured explants [148,157,158]. These studies suggest a role for macrophages in regulating the number of nephrogenic progenitors and vasculogenesis in the kidney. Nevertheless, macrophage-deficient embryos do not exhibit major kidney anatomical phenotypes.

An additional morphogenetic function assigned to heart-specific macrophages is the control the distribution and branching of coronary arteries [61] and the differentiation of the cardiac lymphatic vessels [159]. These functions are supported by the pattern of distribution of macrophages in the developing heart and by the abnormal distribution of coronary vasculature in CSF1-deficient osteopetrotic mice [61]. However, the vascular alteration in the mutant mice occurred during the fetal period of development rather than at initial stages of heart development when programmed cell death occurs [160].

### 7.4. Epimorphic Regeneration

Macrophages, as components of the innate immune defense, are early cellular responders in the repair of injured organs promoting tissue healing and scar formation. A particularly complex response to tissue injury observed in some young, or even adult, vertebrates is their potential to regenerate fully functional anatomical structures following amputation. This process, referred to as “epimorphic regeneration”, is exemplified by the regeneration of fish fins following amputation [161], limb regeneration in urodele amphibians [162], or regeneration of the distal digit tip in postnatal mice [163]. Tissue injury in vertebrate models lacking epimorphic regeneration elicits an inflammatory response, involving the recruitment of neutrophils and macrophages, and is followed by re-epithelization of the wounded skin surface and scar formation. Observation of healing wounds in PU.1-deficient mice reveals that the repair process does not require the involvement of macrophages, as it follows the same temporal sequence in macrophage-deficient and control mice [164]. Unlike this healing process, during epimorphic regeneration, lineage-restricted undifferentiated mesenchymal progenitors are recruited beneath the covering wound epithelium, forming a proliferating blastema that restores the amputated anatomical structures. As observed by [164] during skin wound-healing processes, in the three regenerative models mentioned above (fish fin, salamander limb, and mice digit tip), macrophages are recruited beneath the healing wound surface. However, ablation of macrophages via local application of clodronate liposomes (digit tip and adult axolotl limb) [162,163] or by targeted genetic sensitization to an exogenous cytotoxic poison (NTR-MTZ ablation technology) results in impaired zebrafish fin regeneration [161] or complete inhibition of the mouse digit or axolotl limb [162,163]. Under these conditions, blastemal proliferation is compromised and substituted by fibrous scar tissue. A similar inhibitory effect has been reported during tail regeneration in lizards treated with clodronate liposomes [165].

The mechanism by which macrophages regulate regeneration has not been fully determined, but morphological and genetic analyses indicate that wound healing and regeneration involve an initial pro-inflammatory response that involves macrophages and neutrophils, followed by the accumulation of macrophages with a pro-regenerative phenotype (M2 subtype) [162,166]. In the zebrafish fin model, Wnt/β-catenin signals appear to favor the pro-regenerative phenotype of macrophages [161]. In the regenerating lizard tail, macrophage-derived metalloproteinases have been shown to play a critical role, as their pharmacological inhibition resulted in a complete failure of regeneration [165].

## 8. Conclusions

This review underscores the functional role of macrophages in developing systems, highlighting their contribution to morphogenetic processes. Notably, abnormalities observed in mammals subjected to macrophage ablation are more extensive and pronounced during the postnatal than during the prenatal period. The normal morphogenesis of organs sculpted by programmed cell death (i.e., separated digits), processes accompanied by massive recruitment of macrophages, is illustrative of this feature. In contrast, organs that undergo intense growth and morphogenesis in the postnatal period, such as the mammary gland during puberty and pregnancy, or in epimorphic regenerative processes show more pronounced alterations in adults than in embryos, despite the fact that cellular mechanisms governing their morphogenesis are similar in prenatal and postnatal periods. We propose that this discrepancy might be explained by greater plasticity in the origin of macrophages during embryonic development, which become reduced in postnatal periods, rather than caused by the absence of macrophage trophic functions during organogenesis. Taken together, these findings suggest that in vitro assays, such as organoids or Organ-on-Chip assays (see review by [28]), or explant cultures of embryonic organ primordia, in which the organ is isolated from the rest of the embryo may provide complementary approaches for uncovering previously unrecognized roles of macrophages in embryonic morphogenesis that could remain hidden in the embryo.

## Data Availability

No new data were created or analyzed in this study. Data sharing is not applicable.

## Supplementary Materials

The following supporting information can be downloaded at: https://www.mdpi.com/article/10.3390/cells15151330/s1, Table S1: Summarized phenotypical alterations resulting from macrophage ablation.

## Abbreviations

The following abbreviations are used in this manuscript:

EMPs	Erythromyeloid Progenitors
AGM	Aorta–Gonad–Mesonephros
HSCs	Hematopoietic Stem Cells
CSF1R	Colony-Stimulating Factor-1 Receptor
CSF-1	Colony-Stimulating Factor-1
IL-34	Interleukin 34
TNF-α	Tumor Necrosis Factor
TGFβ	Transforming Growth Factor β
ROS	Reactive Oxygen Species
IGF	Insulin-like Growth Factor
EGF	Epidermal Growth Factor
FGF	Fibroblast Growth Factor
HGF	Hepatocyte Growth Factor
VEGF	Vascular Endothelial Growth Factor
PDGF	Platelet-Derived Growth Factor
ADAMTS	A Disintegrin and Metalloproteinase with Thrombospondin Motifs
MMPs	Matrix Metalloproteinases
CX3CL1	C-X3-C Motif Chemokine Ligand 1
CCL2	C-C Motif Chemokine Ligand 2
MCP-1	Monocyte Chemoattractant Protein-1
NTR/MTZ	Nitroreductase/Metronidazole
