# Supplementary material for: Beyond Immunity: Macrophages as Regulators of Vertebrate Morphogenesis
Source: Cells. 2026 Jul 24;15(15):1330. doi: 10.3390/cells15151330 (PMC13465294; doi:10.3390/cells15151330)
Supplement: Supplementary file 1 [file cells-15-01330-s001.zip › cells-4413474-supplementary.pdf]

Supplementary Table S1. Summarized phenotypical alterations resulting from macrophage ablation. We have excluded hematopoietic alterations that are out of the scope of the present review.

Alteration type	Specie	Time/Type of alteration	Macrophage affected	Phenotypic manifestation	Main phenotype
**Csf-1$^{(op/op)}$**	Mouse	Constitutive germline mutation	Ablation of most tissue-resident macrophages: microglia, osteoclast, ovary, testis, liver, pancreatic	Postnatal period	Osteopetrosis, infertility, delayed growth. Mammary gland, nervous system and pancreatic alterations. Paneth cell deficiencies. Defective angiogenesis
	Rat	Constitutive germline mutation	Ablation of most tissue-resident macrophages: osteoclast, microglia, ovary, testis, liver, pancreatic	Postnatal period	Osteopetrosis, toothless, deficient skeletal growth, infertility, nervous system alteration
**Csf1r $^{(-/-)}$**	Mouse	Constitutive germline mutation	Almost complete depletion of tissue-resident macrophages	Postanal period	More severe phenotype compared with Csf-1(op/op): perinatal lethality, severe osteopetrosis, neurological and developmental abnormalities, no intestinal Paneth cells differentiation, infertility (oocytes alteration), renal tubule alterations and growth impairment
	Rat	Constitutive germline mutation	Almost complete depletion of tissue-resident macrophages	Postanal period	Osteopetrosis, toothless, deficient skeletal growth, infertility, nervous system alteration, loss of visceral white adipose tissue, renal medullary hypoplasia, Peyer´s patches deficiencies, deficient maturation of secondary reproductive organs, deficient growth of mammary gland
	Humans	Hereditary diffuse leukoencephalopathy with spheroids	Microglia	Postnatal period	Progressive dementia
**Il-34 $^{(-/-)}$**	Mouse	Constitutive germline mutation	Microglia and Langerhans cells	Postnatal period	Partial loss of microglia and Langerhans cells. No severe phenotypes
**Pu.1 $^{(-/-)}$**	Mouse	Constitutive germline mutation	Absent	Postnatal period	Hematopoietic failure, neonatal lethality
**NTR/MTZ**	Zebrafish	Larval metamorphosis	Macrophage ablation during larval metamorphosis	Postnatal period	Absence of characteristic pigmented stripes
**Diphtheria toxin**	Mouse	During embryogenesis	Local macrophages	Postnatal period	Persistence of hyaloid vessels
**Clodronate Liposomes**	Mouse	During embryogenesis	Local macrophages	Postnatal period	Persistence of eye hyaloid vessels, inhibition of mouse digit regeneration after a wound
	Axolotl	During embryogenesis	Local macrophages	Epimorphic regeneration	Limb regeneration failure after limb amputation
	Lizard	Adult	Local macrophages	Epimorphic regeneration	Impaired wound healing during tail regeneration
